# Decreased Expression of Sprouty2 in the Dorsolateral Prefrontal Cortex in Schizophrenia and Bipolar Disorder: A Correlation with BDNF Expression

**DOI:** 10.1371/journal.pone.0001784

**Published:** 2008-03-12

**Authors:** Anilkumar Pillai

**Affiliations:** Department of Psychiatry and Health Behavior, Medical College of Georgia, Medical Research Service Line, Veterans Affairs Medical Center, Augusta, Georgia, United States of America; Chiba University, Japan

## Abstract

**Background:**

Current theories on the pathophysiology of schizophrenia suggest altered brain plasticity such as decreased neural proliferation and migration, delayed myelination, and abnormal synaptic modeling, in the brain of subjects with schizophrenia. Though functional alterations in BDNF, which plays important role in neuroplasticity, are implicated in many abnormalities found in schizophrenia, the regulatory mechanism(s) involved in the abnormal signaling of BDNF in schizophrenia is not clear. The present study investigated whether Sprouty2, a regulator of growth factor signaling, is abnormally expressed in schizophrenia, and is associated with the changes in BDNF mRNA in this disorder. The potential effect of antipsychotic drugs on Sprouty2 expression was tested in adult rats.

**Methods and Findings:**

Sprouty2 and BDNF gene expression were analyzed in dorsolateral prefrontal cortex samples from the Stanley Array Collection. Quantitative real-time PCR analysis of RNA in 100 individuals (35 with schizophrenia, 31 with bipolar disorder, and 34 psychiatrically normal controls) showed significantly decreased expression of Sprouty2 and BDNF in both schizophrenia and bipolar disorder. Moreover, a significant correlation between these two genes existed in control, schizophrenia and bipolar subjects. Long-term treatment with antipsychotic drugs, haloperidol and olanzapine, showed differential effects on both Sprouty2 and BDNF mRNA and protein levels in the frontal cortex of rats.

**Conclusion:**

These findings demonstrating decreased expression of Sprouty2 associated with changes in BDNF, suggest the possibility that these decreases are secondary to treatment rather than to factors that are significant in the disease process of either schizophrenia and/or bipolar disorder. Further exploration of Sprouty2-related signal transduction pathways may be helpful to design novel treatment strategies for these disorders.

## Introduction

Abnormal neurodevelopment (decreased neural proliferation and migration, delayed myelination, abnormal synaptic modeling) and subsequent enhanced vulnerability to untreated illness are implicated in the pathophysiology of schizophrenia [1]–[3]. Neurotrophic factors exert important actions on the development, maintenance, and function of the peripheral and central nervous system [4]–[7]. For example, deficits in brain derived neurotrophic factor (BDNF) signaling are implicated in many abnormalities found in schizophrenia [8]–[9]. Postmortem studies showed changes in BDNF as well as its receptor, TrkB expression in different brain areas of subjects with schizophrenia [10]–[15]. Although several studies indicated altered serum BDNF levels in subjects with schizophrenia [16]–[18], some other studies failed to find any difference in serum BDNF levels between drug-naïve schizophrenia and control subjects [19]–[20]. This discrepancy may be due to factors such as biological heterogeneity of the subjects, duration of illness or the antipsychotic medications. More recently, we found a significant reduction in plasma BDNF levels in subjects with first-episode psychosis in comparison with normal healthy controls, and plasma BDNF levels negatively correlated with positive symptom scores at base line [21]. In addition to the clinical observations, pre-clinical studies showed that antipsychotic drugs, the primary choice of treatment for schizophrenia, exert their beneficial effects through growth factor-mediated signaling pathways [22]–[23]; however, the regulatory mechanism(s) involved in the abnormal signaling of BDNF in schizophrenia is not clear.

Recent studies indicate the role for Sprouty (Spry) proteins in growth factor signaling [24]. Sprouty was first identified in Drosophila and shares a high degree of evolutionary sequence homology in the C-terminus, although mammalian Spry differs from Drosophilia Spry within the N-terminus. Growth factors, including BDNF, induce Sprouty2 (Spry2, a member of sprouty family) expression at the transcriptional level and phosphorylate Spry2 on critical tyrosine residues, suggesting regulation at the transcriptional and the post-translational level [24]–[25]. Growth factor-mediated receptor-tyrosine kinase (RTK) activation leads to the phospholipid-dependent translocation of Spry2 to plasma membrane, where it binds to various signaling molecules such as Grb-2 [24]. Although Spry proteins inhibit RTK signaling induced by many growth factors, including BDNF, EGF-mediated extracellular signal-regulated kinase (ERK)/mitogen-activated protein kinase (MAPK) signaling is augmented in a cell-type dependent manner [25]. *In vitro* studies showed that overexpression of Spry proteins antagonizes proliferation, migration and differentiation in response to various growth factors [24]–[25]. In addition, a recent study showed that Spry2 was involved in the development of the CNS by inhibiting both neuronal differentiation and survival through a negative-feedback loop that downregulates BDNF-mediated signaling pathways [26].

Considering neurotrophin involvement in the neuropathogenesis of schizophrenia, the possible role of Spry2, a prototypical member of the sprouty family, was investigated in schizophrenia. The expression of BDNF and Spry2 was examined by quantitative real-time PCR (qRT-PCR) in the Stanley Array Collection, derived from dorsolateral prefrontal cortex (DLPFC) of individuals with schizophrenia, bipolar disorder, or psychiatrically normal controls. The study also examined whether the changes in Spry2 mRNA correlated with the changes in BDNF mRNA; changes which may influence the pathophysiology and pharmacological treatment of schizophrenic patients.

## Results

### Sprouty2 expression is decreased in schizophrenia and bipolar disorder, and correlates with BDNF expression

Reverse-transcription real-time PCR was used for the determination of mRNA expression levels of Spry2 and BDNF for the postmortem samples. As shown in Table 1, no significant correlation was found between the mRNA expression of either Spry2 or BDNF and the confounding variables, such as age at death, PMI, brain pH, brain weight, refrigeration interval, gender, hemisphere, smoking status at time of death, age of onset, duration of illness, lifetime alcohol use, or lifetime substance abuse. Furthermore, there was no effect of suicide or cardiac death on the mRNA expression of Spry2 or BDNF (data not shown).

**Table 1:** Correlations between BDNF or sprouty2 mRNA levels and confounding variables

Variable	Control				Schizophrenia				Bipolar disorder			
	BDNF		Sprouty2		BDNF		Sprouty2		BDNF		Sprouty2	
	r^a^	p^b^	r	p	r	p	r	p	r	p	r	p
Age	−0.085	0.587	−0.100	0.236	0.244	0.188	0.088	0.608	−0.022	0.899	−0.166	0.481
PMI	−0.012	0.482	−0.075	0.673	0.185	0.287	0.017	0.922	0.234	0.206	−0.277	0.132
Brain Ph	0.097	0.584	0.057	0.747	0.182	0.296	0.159	0.359	−0.183	0.325	0.127	0.494
Brain weight	0.082	0.645	0.019	0.916	0.289	0.092	0.069	0.694	0.335	0.065	0.169	0.362
Refrigeration interval	−0.127	0.431	−0.153	0.488	0.099	0.365	0.086	0.528	−0.121	0.263	0.083	0.611
Gender	−0.077	0.512	0.081	0.711	0.022	0.878	−0.033	0.854	0.070	0.692	−0.118	0.527
Hemisphere	0.028	0.765	−0.095	0.634	−0.123	0.332	0.077	0.718	0.199	0.261	−0.184	0.447
Smoking at the time of death	−0.145	0.521	−0.293	0.186	−0.207	0.279	−0.230	0.229	0.151	0.491	−0.035	0.870
Age of onset					0.367	0.060	−0.165	0.344	0.074	0.691	0.021	0.909
Duration of illness					−0.243	0.160	0.332	0.051	−0.299	0.102	−0.031	0.099
Lifetime alcohol use	0.111	0.532	−0.106	0.550	−0.109	0.534	0.023	0.896	0.265	0.126	0.093	0.622
Lifetime substance use	0.067	0.707	0.163	0.358	−0.096	0.600	−0.067	0.709	0.199	0.282	0.163	0.381

a Correlation value

b significance, p value from ANOVA

Pearson's product moment correlation shown for age, PMI, brain pH, brain weight and refrigeration interval.

Kendall's rank correlation tau shown for gender, hemisphere, smoking status, age of onset, duration of illness, lifetime alcohol use and lifetime substance abuse.

Next, the possible relationship between the effect of antipsychotic medication and the mRNA expression of Spry2 or BDNF was examined. The diagnosis status table obtained from Stanley Foundation on the patients and control subjects indicated that all schizophrenia patients were treated with antipsychotic drug(s) at some point during their disease condition. In contrast, a significant number of patients in bipolar disorder group did not previously receive (n = 12 of 31) antipsychotic medications. No significant correlation was found between antipsychotic use at time of death and Spry2 or BDNF mRNA expression in bipolar patients (Table 2). To understand further the effect of antipsychotic medication on Spry2 and BDNF, the mRNA levels of spry2 and BDNF were examined in antipsychotic-naive patients with bipolar disorder. Spry2 expression was significantly low (p = 0.037) in antipsychotic-naive patients with bipolar disorder (n = 12) compared to control subjects. Although mean BDNF mRNA levels were decreased by 36% in antipsychotic-naive patients with bipolar disorder, this decrease did not reach statistical significance (p = 0.052). As indicated in Table 2, no significant correlations were found between levels of lifetime antipsychotic exposure and expression of either Spry2 or BDNF in schizophrenia group.

**Table 2:** Effect of antipsychotic treatment on BDNF and sprouty2 mRNA expression

Variable	Group	BDNF		Sprouty2	
		Correlation	p value	Correlation	p value
Antipsychotic use at the time of death^a^	Bipolar	−0.176	0.457	−0.261	0.265
Lifetime antipsychotic exposure^b^	Bipolar	−0.178	0.346	−0.246	0.189
Lifetime antipsychotic exposure^b^	Schizophrenia	−0.202	0.244	−0.255	0.139

a Kendall's rank correlation.

b Spearman's correlation.

Lifetime antipsychotic dose in fluphenazine milligram equivalents.

p values are from ANOVA.

**Figure 1:**
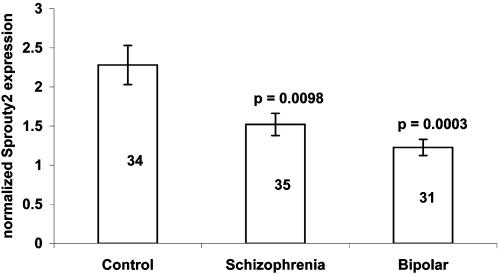
Normalized Sprouty2 mRNA expression in schizophrenia, bipolar and control subjects. Values are expressed as mean±SEM. The number of individuals per sample is indicated within each bar. Sprouty2 expression is significantly low in schizophrenia and bipolar subjects as compared to control subjects. Level of significance as compared to control subjects is shown above each bar.

**Figure 2:**
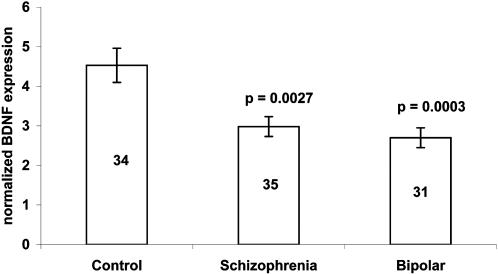
Normalized BDNF mRNA expression in schizophrenia, bipolar and control subjects. Values are expressed as mean±SEM. The number of individuals per sample is indicated within each bar. BDNF expression is significantly low in schizophrenia and bipolar subjects as compared to control subjects. Level of significance as compared to control subjects is shown above each bar.

A significant reduction in mean Spry2 mRNA levels was observed in the schizophrenia (33%; p = 0.0098) and bipolar groups (46%; p = 0.003) compared to the control group (Figure 1). Similarly, mean BDNF mRNA levels were significantly reduced in schizophrenia group (34%; p = 0.0027) and bipolar group (40%; p = 0.003) as compared to the control group (Figure 2). To study the relationship between Spry2 and BDNF, Pearson's correlation was performed in schizophrenia group, bipolar group and control group (Table 3). Significant high correlation between Spry2 and BDNF mRNA expression levels was found in schizophrenia group (p = 0.01), bipolar group (p = 0.02) and control group (p = 0.016).

**Table 3:** Correlation between BDNF and sprouty2 mRNA levels

Group	Correlation^a^	p value
Control	0.407	0.016
Schizophrenia	0.414	0.010
Bipolar	0.400	0.020

a Pearson's correlation.

### Antipsychotic drugs modulate BDNF and Sprouty2 expression in rat frontal cortex

**Figure 3:**
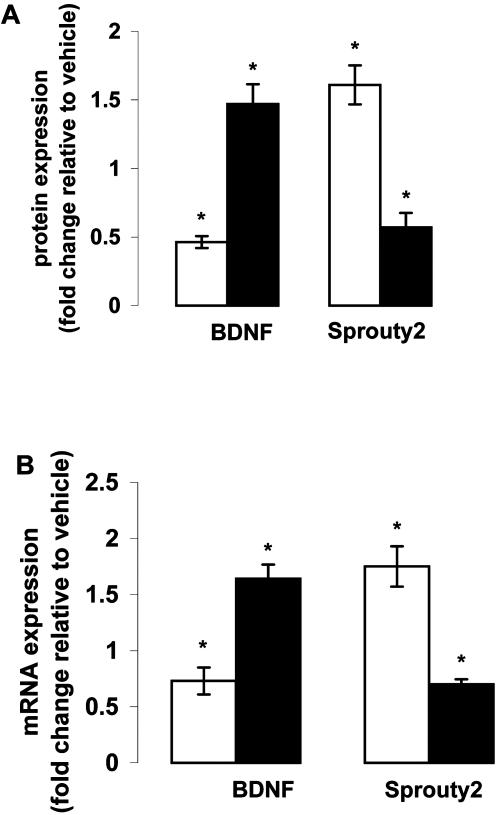
BDNF and Sprouty2 expression in the frontal cortex of rats treated with haloperidol or olanzapine. Adult rats were treated with haloperidol (2 mg/kg) or olanzapine (10 mg/kg) through drinking water for 45 days, and (A) protein and (B) mRNA levels of BDNF and Sprouty2 in frontal cortex were estimated after the treatment. BDNF protein levels were measured by ELISA and Sprouty2 proteins levels were estimated by Western blot analysis. mRNA levels of both BDNF and Sprouty2 were estimated by qRT-PCR analysis. Open bars represent haloperidol-treated rats whereas filled bars represent olanzapine-treated rats. Values are expressed as fold change relative to vehicle-treated rats. *p<0.05, n = 10–12 per group.

In order to test the potential effect of antipsychotic medication, we evaluated mRNA as well as protein levels of BDNF and Spry2 in the frontal cortex of rats treated with haloperidol and olanzapine. Haloperidol treated rats showed significant reduction in mRNA (p = 0.03) as well as protein (p = 0.019) levels of BDNF, where as olanzapine treated rats showed significant increase in mRNA (p = 0.023) as well as protein (p = 0.045) levels of BDNF as compared to vehicle-treated rats (Figure 3). Interestingly, significant increase in Spry2 mRNA (p = 0.027) and protein (p = 0.02) expression was found in the frontal cortex of haloperidol-treated rats whereas olanzapine-treated rats showed significant decrease in Spry2 mRNA (p = 0.018) and protein (p = 0.041) expression as compared to vehicle-treated rats.

## Discussion

The present study for the first time shows that the expression of Spry2, a key regulator of growth factor signaling, is altered in schizophrenia and bipolar disorder compared with normal controls. The key findings from the present study are: First, gene expression of Spry2 and BDNF, a known inducer of Spry2, are down-regulated in the DLPFC of schizophrenia and bipolar subjects. Second, a significant positive correlation was observed between Spry2 and BDNF in schizophrenia, bipolar and normal subjects. Third, antipsychotic drug treatments showed differential effects on mRNA as well as protein levels of Spry2 and BDNF in rat frontal cortex.

The present study was conducted using the RNA samples from DLPFC area of schizophrenia, bipolar and normal subjects. Evidence from neuropsychological, neuroimaging, histopathological, and neurochemical studies implicate the role of DLPFC in the pathophysiology of schizophrenia [27]. In addition, a number of studies have used the same set of samples obtained from Stanley Array Collection to examine various pathological markers in schizophrenia and bipolar disorder [28]-[30]. Though there are a number of potential variables (age, PMI, brain pH, history of substance abuse, cause of death, alcohol use, or smoking), which can affect the quality of RNA as well as the gene expression status, the results from the present study did not show any significant correlation between BDNF or Spry2 mRNA levels and these confounding variables.

Spry2 is the prototypical member of a new family of RTK-signaling modulators, Sprouty proteins. The data from the present study suggest that Spry2 gene expression is significantly decreased in patients with either schizophrenia or bipolar disorder. It has been previously reported that BDNF stimulates Spry2 expression in neurons, and CREB and Sp1 are the two transcription factors that regulate Spry2 promoter activity in response to BDNF [26]. To determine whether BDNF is involved in the mechanism that contributed to decreased Spry2 expression in schizophrenia and bipolar subjects, the present study examined the correlation between the mRNA expression levels of Spry2 and BDNF. The changes in Spry2 mRNA expression were significantly correlated with BDNF mRNA expression in schizophrenia, bipolar and normal subjects suggesting the importance of BDNF in the regulation of Spry2 expression. However, further studies are needed to establish whether reduced expression of BDNF is sufficient to produce the altered expression of Spry2 in the DLPFC of subjects with schizophrenia or bipolar disorder since other growth factors, such EGF and FGF are also known to regulate Spry2 expression, at least in *in vitro* conditions [31]–[33], and the transcription factors CREB and Sp1 are involved in other growth factor signaling pathways [34]–[35].

The data from antipsychotic-treated rats showed significant effect of haloperidol and olanzapine on Spry2 mRNA and protein levels in frontal cortex. The decrease in Spry2 expression after haloperidol treatment might be due to the adverse side effects of haloperidol after long-term treatment. It has been shown previously that neurotoxic stresses lead to reduction of Spry2 expression in mature neurons and the reduced Spry2 protein levels were significantly restored by BDNF treatment [26]. Long-term haloperidol-treatment has been shown to increase neurotoxicity markers, including pro-apoptotic proteins [36]–[37], and decrease BDNF protein levels in rat frontal cortex [37]. In addition, compounds which can up-regulate BDNF signaling pathway have been shown to prevent haloperidol treatment-induced neuronal apoptosis [37]. Spry2 protein levels were decreased in rat frontal cortex after olanzapine treatment with a parallel increase in BDNF protein levels. Since Spry2 is a known inhibitory molecule of cell proliferation and survival [24]–[25], data on decreased Spry2 expression after olanzapine treatment is in agreement with the earlier reports that olanzapine treatment increases cell proliferation in rat frontal cortex [38]–[39]. Again, as in haloperidol-treated rats, the olanzapine-treated rats also showed an inverse relationship between BDNF and Spry2 expression. It is important to note that Spry2 expression can be induced upon growth factors such as EGF, FGF and BDNF [26], [31]–[33], whereas its expression can be downregulated by TGF-β1 [40]. Therefore, the changes observed in Spry2 expression would be the net effect of multiple growth factor signaling pathways. Interestingly, rodent studies have indicated that the beneficial effects of antipsychotic drugs are mediated through growth factor signaling pathways [22]–[23]. This implies that the changes in Spry2 expression with antipsychotic-treatment may involve additional mechanisms other than BDNF signaling pathway.

The Spry2 gene regulates RTK signaling of many molecules such as EGF, EGF, VEGF and BDNF. These growth factors as well as neurotrophins play important roles in neuronal development and plasticity. Spry2 is of particular interest as a downstream target gene as well as a regulator for BDNF signaling. BDNF plays an important role in regulating dopaminergic, glutamatergic, and other neuronal signaling pathways, alterations of which are observed in schizophrenia [41]–[42]. Moreover, postmortem studies using brain samples from different cohorts showed changes in BDNF expression as well as the expression of its receptor, TrkB, in different brain areas of subjects with schizophrenia [10]–[15]. There are a number of other RTK-binding molecules in addition to BDNF, which are implicated in schizophrenia. For example, neuregulin 1 *(*NRG1*)* is a potential susceptibility gene for schizophrenia [43], and alterations in NRG1-ErbB signaling occur in the prefrontal cortex of schizophrenic patients [44]–[45]. NRG1-ErbB signaling regulates oligodendrocyte development and myelination [44], [46] and abnormalities in oligodendroglia and myelin are reported in schizophrenia [47]. In addition, the role of EGF has been investigated recently in the pathophysiology of schizophrenia. Although one study reported abnormal expression of EGF and its receptor in the forebrain and serum of subjects with schizophrenia [48], another study could not find any significant change in the serum EGF levels in schizophrenia subjects compared to controls indicating the possibility that EGF may serve as a state marker, i.e., as an index of symptom-linked deficits [49]. A recent study showed that knockdown of endogenous Disrupted-in-schizophrenia 1 (DISC1), a putative susceptibility gene for psychoses such as schizophrenia and bipolar disorder, by small interfering RNA in cortical neurons suppressed phosphorylation of ERK, but the exact mechanisms remain unknown [50]. These studies indicate the possible involvement of Spry2 in the function of many genes currently implicated in schizophrenia.

Growth factors function through RTKs and activate Ras-ERK MAPK signaling to regulate numerous cellular events [51]. ERK pathway has been implicated in regulation of neuronal function, cell survival and in learning and memory [52]. Spry2 specifically inhibits Ras-ERK MAPK signaling, leaving other MAPK and PI3K pathways unaffected [31], [53]. Spry2 exerts differential effects on RTK-mediated signaling pathways at the level of Ras or Raf, depending upon the cell type and growth factor [24]–[25], [28]. Spry2 inhibits FGF-induced ERK activation by interfering with FRS2/Shp2/Grb2 interactions [54], whereas it activates EGF-mediated ERK activation by preventing c-Cbl-mediated ubiquitination and endocytosis of activated EGFR [55]. Furthermore, a recent study showed that the reduced level of Spry2 induced by TGF-β1 is associated with inhibition of ERK activation in response to EGF stimulation [40]. The available literature on ERK signaling in postmortem brain samples from schizophrenia indicate a significant increase in ERK2 protein as well as mRNA levels in cerebellum samples from schizophrenia subjects [56]. But, accumulating evidence from rodent studies indicate differential effects of antipsychotic drugs on ERK signaling in brain [57]–[59]. Therefore, additional studies are needed to correlate Spry2 expression and ERK signaling in schizophrenia, which will help to identify the downstream signaling pathways regulated by Spry2 in schizophrenia.

Taken together, our data from postmortem as well as animal studies indicate that the altered expression of Spry2 may be secondary to antipsychotic treatment rather than to factors that are significant in the disease process of either schizophrenia and/or bipolar disorder. The lack of correlation between the expression of Spry2 and antipsychotic medication at the time of death suggest that additional studies using more number of subjects with/without antipsychotic medication would be of great interest. Further exploration of Spry2-related signal transduction pathways will be helpful to design novel treatment strategies for schizophrenia and bipolar disorder.

## Materials and Methods

### Human Postmortem Samples

RNA samples from postmortem brain specimens, Brodmann's area 46 (in the DLPFC), from 35 individuals with schizophrenia, 31 individuals with bipolar disorder, and 34 psychiatrically normal controls were obtained from the Stanley Array Collection of the Stanley Brain Collection. A description of the Stanley Brain Collection and more detailed demographics and samples' quality were previously reported [60]. RNA expression analysis was conducted with the samples coded to keep investigators blind to diagnostic status. At the time of sample decoding, diagnostic status and a range of clinical variables were provided for analysis (see Table 4).

**Table 4:** Demographic data for post mortem brain samples.

Variable	Control (N = 34)	Schizophrenia (N = 35)	Bipolar (N = 31)
Age (years, mean±SD)	48.82±7.57	42.57±8.46	44.93±10.99
Gender (male/female)	25/9	26/9	15/16
PMI (h, mean±SD)	29.47±13.04	31.4±15.54	36.61±18.11
Refrigeration interval (h, mean±SD)	3.71±2.60	5.97±4.22	8.74±7.22
Brain pH (mean±SD)	6.61±0.27	6.47±0.24	6.46±0.28
Brain weight (g, mean±SD)	1443.21±150.54	1442.11±107.49	1410.64±135.36
Age of onset (years, mean±SD)	0	21.28±6.07	24.77±8.95
Duration of illness (years, mean±SD)	0	21.28±10.14	20.16±9.88
Hemisphere (left/right)	16/18	17/18	18/13
Lifetime alcohol use	16	25	27
Lifetime drug abuse	5	21	22
History of psychosis	0	35	18

### RNA quantification

qRT-PCR was performed on a SmartCycler (Cepheid, Sunnyvale, CA) using a SuperScript III Platinum SYBR Green One-Step qRT-PCR kit (Invitrogen, Carlsbad, CA). Master mixes were prepared and used in the PCR amplifications. A typical reaction of a total volume of 25 µl consisted of 0.5 µl Superscript III RT/Platinum Taq mix, 12.5 µl 2X SYBR Green Reaction Mix (includes 0.4 mM of each dNTP and 6 mM MgSO4), 12.5 pMol of each of forward or reverse primers and 5 µl DEPC-treated water. Approximately 0.5–1.0 µg RNA was used as template. PCR amplification was done with an initial incubation at 55°C for 120 sec, then at 95°C for 120 sec followed by 35 cycles of 95°C for 15 sec, 50°C for 30 sec, 72°C for 30 sec and final melting curve from 55°C to 95°C with 0.2C/sec. The standard curve used for determining the relative quantity in each sample was constructed by the amplification of serial dilutions of cDNA. Primer specificity was confirmed by melting curve analysis and electrophoresis of PCR products on a 2% agarose gel to confirm the presence of a single band of the predicted size. All measurements were performed in triplicates. The data were normalized to three control genes (glyceraldehyde 3-phosphate dehydrogenase (GAPDH), *ß*-2-microglobulin (β2-MG) and *ß*-actin) and a geometric mean of these genes. Primers utilized are listed in Table 5.

**Table 5:** List of primer sequences used for postmortem studies.

	Gen Bank accession No	Forward	Reverse	Base pairs
**BDNF**	NM_170735	5′-AAACATCCGAGGACAAGGTG-3′	5′-AGAAGAGGAGGCTCCAAAGG-3	634-883
**Sprouty2**	NM_005842	5′-CCCCTCTGTCCAGATCCATA-3′	5′- CCCAAATCTTCCTTG CTCAG-3′	695-896
**GAPDH**	NM_002046	5′-GAGTCAACGGATTTGGTCGT-3′	5′-TTGATTTTGGAGGGATCTCG-3′	122-359
**β2-MG**	NM_004048	5′-CCAGCGTACTCCAAAGATTCA-3′	5′-TGCTCCACTTTTTCAATTCTCTC-3′	123-201
**β-actin**	NM_001101	5′-GGACTTCGAGCAAGAGATGG–3′	5′-AGCACTGTGTTGGCGTACAG-3′	736-969

### Animal experiments

#### Animals

Adult male albino rats (weighing 225–250 g; Harlan Sprague–Dawley, Inc., Indianapolis, IN, USA) were housed singly under a 12-h light/12-h dark cycle and at a constant temperature (25°C) and humidity, and allowed free access to food and water. Animal use procedures were performed after being reviewed and approved by Medical College of Georgia Committee on Animal Use for Research (CAURE) and Veterans Affair Medical Center Subcommittee on Animal use. The procedures were consistent with Association for Assessment and Accreditation of Laboratory Animal Care (AAALAC) guidelines as per Public Health Service Policy on Humane Care and Use of Laboratory Animals.

#### Drug treatment

Haloperidol (Sigma Chemical Company, St Louis, MO, USA) and olanzapine (A & A Pharmachem; Toronto, ON, Canada) were dissolved in a 0.1-M acetic acid and subsequently diluted (1∶100) with tap water to administer the final daily dose of drug. All drugs were prepared daily and administered in solutions, which replaced drinking water. The amount of drug intake was measured every day and adjustments were made depending upon the fluid consumed and weight of the animals. This method was preferred over multiple intramuscular injections to maintain more constant drug levels and to reduce stress and neuromuscular damage. Rats (n  = 10–12 per group) received haloperidol (2 mg/kg/day) or olanzapine (10 mg/kg/day) for 45 days. The haloperidol dose was chosen based on previous studies [23], in which it was found to result in plasma levels comparable to therapeutic plasma levels in humans. This dose is also comparable to the dose optimum for pharmacological effects [61]–[63]. The choice of olanzapine, a representative atypical antipsychotic, and its dose and duration of treatment was based on their different pharmacological and clinical profiles, and neurochemical effects reported in a large number of earlier studies [23], [64]–[66]. Tap water containing 0.1 M acetic acid was used for the control group to assure that an unanticipated effect of the vehicle was not present.

### RNA quantification in rat frontal cortex samples

Frontal cortex samples from vehicle and antipsychotic-treated rats were collected and RNA was extracted using a commercially available kit (SV RNA Isolation, Promega, Madison, WI), according to manufacturer's instructions. mRNA was measured using a SuperScript III Platinum SYBR Green One-Step qRT-PCR kit as described earlier for human tissue with the following primers: Sprouty2: (629–811 base pairs, Gen Bank accession No: NM_011897, forward primer 5′-GGGTCTCGGAGCAGTACAAGG-3′; reverse primer 5′-GTAGGCATGCAGACCCAAAT-3′), BDNF (1115-1363 base pairs, Gen Bank accession No: NM_007540, forward primer 5′-GCGGCAGATAAAAAGACTGC-3′; reverse primer 5′- CTTATGAATCGCCAGCCAAT -3′) and housekeeping gene, ribosomal protein S3 (RPS3) (284-466 base pairs, Gen Bank accession No: NM_012052, forward primer 5′-AATGAACCGAAGCACACCATA-3′; reverse primer 5′-ATCAGAGAGTTGACCGCAGTT-3′).

### Protein quantification in rat frontal cortex samples

Frontal cortex samples were collected and homogenized in ice-cold buffer (10 mM Tris-HCl, pH 7.5, 150 mM NaCl, 0.1% SDS, 1% Nonidet P-40, 1% sodium deoxycholate) supplemented with protease inhibitor cocktail (Sigma) containing 104 mM AEBSF, 0.08 mM aprotinin, 2 mM leupeptin, 4 mM bestatin, 1.5 mM pepstatin A, and 1.4 mM E-64. After 15-min incubation on ice, the extracts were clarified by centrifugation at 14,000 rpm for 15 min at 4°C and stored at –70°C. Protein concentrations were determined by the bicinchoninic acid method (BCA Protein Assay Kit, Sigma). Equal amounts of protein were resolved in SDS-polyacrylamide gels and transferred electrophoretically onto a nitrocellulose membrane (Bio-Rad, Hercules, California, United States). Membranes were blocked for 1 h in TBST (10mM Tris-HCl, pH 8.0, 138 mM NaCl, 2.7 mM KCl, and 0.05% Tween-20) and 5% non-fat milk and incubated overnight with a rabbit antibody to sprouty2 (Santa Cruz Biotechnology, Santa Cruz, California, United States) and visualized using ECL (Pierce, Rockford, Illinois, United States) with a secondary antibody coupled to horseradish peroxidase. For actin normalization, blots were incubated in strip buffer (62.5 mM Tris-HCl [pH 7.4], 2% SDS, and 90 mM β-mercaptoethanol) at 65°C for 20 min with agitation, followed by probing with a rabbit antibody to β-actin (the antigen corresponds to amino acids 20–33, conserved in all actin isoforms) as described above. To ensure that quantification used an exposure that was within the linear range of the film used to image the blot, a series of images with increasing exposure time to the blot were developed. The intensity of the bands on each image was quantified using Quantity one software (Bio-Rad) after normalizing to the expression of β-actin.

### Brain-derived Neurotrophic Factor (BDNF) Immunoassay

BDNF protein was measured with a conventional sandwich ELISA using the BDNF Emax immunoassay system (Promega, Madison, WI, USA) according to the protocol of the manufacturer.

### Statistical analysis

The mean quantity value for Spry2 or BDNF mRNA for each individual was normalized to the geometric mean of the three housekeeping genes to obtain the relative amount of each transcript in each individual. Continuous variables (age at death, postmortem interval, brain pH, brain weight and refrigeration interval) were tested by regression; dichotomous variables (gender, brain hemisphere analyzed, smoking status at time of death, age of onset, duration of illness, and history of exposure to antipsychotic medication) and ordinal variables (lifetime alcohol use and lifetime substance abuse) were tested by ANOVA. The strength of the association with Spry2 or BDNF expression was measured with Pearson's product moment correlation for continuous variables and Kendall's rank correlation for binary and ordinal variables. Effect of exposure to antipsychotics on Spry2 and BDNF mRNA as well as protein levels in rats were analyzed by one-way ANOVA followed by post-hoc Dunnett test with comparison to the vehicle-treatment group. Differences in means between groups were considered significant if p<0.05. SPSS version 14.0 software was used for statistical analyses.
